# Penn Access Summer Scholars program: a mixed method analysis of a virtual offering of a premedical diversity summer enrichment program

**DOI:** 10.1080/10872981.2021.1905918

**Published:** 2021-03-31

**Authors:** Cecilia Zhou, Chielozor Okafor, Jamal Hagood, Horace M. DeLisser

**Affiliations:** Program for Diversity and Inclusion, Academic Programs Office, Perelman School of Medicine, University of Pennsylvania, Philadelphia, PA, USA

**Keywords:** Healthcare disparities, healthcare workforce diversity, medical student diversity, medical student admissions, minority pre-medical summer programs, pipeline programs, underrepresentation in medicine

## Abstract

In the USA, numerous summer programs are available for undergraduate students that seek to increase the number of individuals from groups underrepresented in medicine (URM) that matriculate to medical school. These programs have typically been conducted at research-focused institutions, involving hands-on-research and various enrichment experiences. For 2020, the COVID-19 pandemic resulted in the suspension of on-campus student activities at American universities, necessitating a switch to a virtual format for these URM-focused programs. Outcomes, however, from these programs conducted virtually, necessitated by the COVID-19 pandemic, have not been reported. The Penn Access Summer Scholars (PASS) program at the Perelman School of Medicine (PSOM) targets URM undergraduates, providing two consecutive summers of mentored research and enrichment experiences, with the goal of enabling participants’ matriculation to PSOM. PASS has been an 8 week on-campus experience, but during summer 2020, virtual programming of 6 weeks was provided due to the COVID-19 pandemic. Participants in the 2020 virtual offering of PASS completed pre- and post-program surveys that included 5-point Likert-style and open-ended questions to determine the impact of the programing on self-assessments of research skills, familiarity with the physician identity, and preparedness to be a PSOM student. Post-program, participants also assessed program administration and content. With respect to program objectives, participants reported significant increases in their self-reported confidence in conducting research, understanding of physician identity, and sense of preparedness for medical school. The educational value of the program content, their level of engagement in the program and the overall quality of the program were rated as excellent or outstanding by large majorities of respondents. Content analyses of participant comments were consistent with these quantitative results. Therefore, a premedical summer enrichment program targeting URM undergraduates can be successfully conducted virtually to achieve program objectives and may increase the availability to these initiatives.

## Introduction

Diversity in medical education and in the physician-workforce has been recognized as both societal and healthcare imperatives [1–5]. This has prompted, over the last 30 years, the establishment of summer programs that target undergraduate students from historically disadvantaged groups to expose them to research and other enrichment experiences with the goal of enhancing participants’ competitiveness for and/or increasing participant success in medical school [6–14]. These programs are varied, but broadly have been offered either as efforts to increase the national pool of medical school applicants from groups underrepresented in medicine (URM) or as a part of pipeline or early-assurance admission programs. Although there is evidence of the effectiveness of these programs [15,16], the costs associated with the onsite, in-person format of these programs represent a potential barrier to the establishment of more URM-focused pre-medical summer enrichment programs.

To support its efforts in recruiting a diverse student body, the Perelman School of Medicine (PSOM) at the University of Pennsylvania, since 2008, has sponsored the Penn Access Summer Scholars (PASS) program. The program targets URM and first-generation and/or low-income (FGLI) undergraduate students, providing two summers of mentored research, didactic presentations and enrichment activities, with the goal of enabling participants’ successful matriculation to PSOM.

Typical of programs of this type, PASS has been an on-campus experience, with housing provided and participants interacting directly with peers, mentors, lab personnel and program leaders. However, because of the COVID-19 pandemic, all on-campus, in-person activities were suspended by the University of Pennsylvania for the summer of 2020 for all university and visiting students. This necessitated the reconfiguration of PASS as a virtual program. Here, we describe the initial outcomes from our delivery of PASS as a virtual experience, findings that may have implications for increasing the availability of these types of summer enrichment programs to more students.

## Methods

### Overview the PASS program

The PASS program was launched in 2008 by the Office of Admissions of PSOM as an initiative to increase the diversity of students that matriculated to its medical school. It was originally conceived of as a program in which disadvantaged students (based on race, ethnicity and/or socioeconomic status) from partnering colleges and universities would spend two consecutive summers after their sophomore and junior years of college on the campus of the University of Pennsylvania doing mentored research and participating in pre-medical enrichment experiences. Students who successfully completed the two summers of PASS would be eligible to apply to PSOM without having to submit a MCAT score. Beginning with 3 partnering schools – Haverford College, Princeton University, and the University of Pennsylvania – the program has expanded to now include 6 additional institutions – Bryn Mawr College, Howard University, Morehouse College, Oakwood University, Spelman College, and Xavier University of Louisiana. As of September 2019, 50 students (15 males and 35 females) had participated in the two summers of program. Of this group, 38 had matriculated to PSOM, 8 gained admission to another medical school, 1 had deferred application to medical school to conduct research, and 3 opted not to pursue a career as a physician. For the on-campus programing, students currently receive a stipend of 4000 USD and are provided housing (~ 1500 USD/student) free-of-charge. Funding for the program has come from institutional funds and extramural grants from the National Institutes of Health.

### Description of the virtual PASS program

#### Development of the virtual program

The established on-campus program has involved two consecutive, 8-week summer experiences, the goals of which have been to:

- Promote the development of physician identity and professionalism in the PASS participants
- Expose PASS participants to high quality biomedical research as well as develop pertinent research skills
- Foster the mentor/mentee relationship between the PASS participants and their supervising research faculty
- Create a sense of community and cohesion among the cohort of PASS participants
- Facilitate the participants’ transition into PSOM

It has involved 40 hours/week of mentored research (25 hours) with various enrichment events and experiences (15 hours) that include a weekly combination of didactics, physician shadowing, visits to student-led community clinics, physician career narratives, book club discussions, events with medical students and one-on-one meetings with program leadership.

In adhering to institutional mandates, the PASS program for summer 2020 was delivered as a 6-week experience in a virtual format, while still pursuing the five goals noted above. The virtual program was also a 40 hours/week experience for the participants, but in contrast to the on-campus experience, it was structured to be 15 hours/week of research-focused activities with 25 hours of enrichment recapitulating as much as possible the enrichment programing that was typically provided. In addition, the virtual program was modified to have two paid rising second-year medical students to help coordinate and facilitate the online experience for the PASS participants. The medical student coordinators also assisted in content development and program assessments. Comparisons of the on-campus versus the virtual offerings of PASS are summarized in Table 1.

**Table 1.** PASS on-campus versus virtual programing

PASS Program	Number of Weeks	Number of Hours/week	Mentor Research Activities	Program of Enrichment (Hours/week)	Near-Peer Mentoring
On-Campus (Usual program)	8	40	25	15	No
Virtual (Summer 2020)	6	40	15	25	Yes

#### Components of the virtual programing

The 2020 virtual PASS programing included four major components that are detailed in Table 2. These involved a virtual research experience with students conducting research remotely; a portfolio of enrichment experiences that included various facilitated group discussions, physician interactions, didactics and special events; near-peer mentoring and facilitation provided by the medical student coordinators; and individualized advising and coaching by the program team. The students’ schedule (in Eastern Standard Time) was as follows:

- 9:00 AM-1:00 PM: Research-related activities, preparation for future sessions and/or lunch
- 1:00 PM – 3:30 PM: Enrichment and didactic activities done virtually as a group
- 3:30 PM −5:00 PM: Flexible time for 1-on-1 meetings with program team and for preparation

**Table 2.** PASS program components and their elements

Program Components	Elements of Components	Comments
Research	1. Virtual mentored research	Included participation in virtual lab/research-related meetings, journal clubs, and conferences
	2. Weekly 1-on-1 meetings with research mentor	Focused on developing the mentor-mentee relationship
	3. Weekly online research modules	Topics included responsible conduct of research, reading a research article, creating a journal club presentation, designing a research poster, and communicating professionally
	4. Research presentations	This included work-in-progress and journal club presentations to PASS peers
Enrichment	1. Podcast discussions	Podcasts were drawn from a variety of sources and addressed topical issues in medicine, science and society; discussions were facilitated by the medical student coordinators
	2. Physician career narratives	Narratives were provided by physicians from diverse backgrounds and clinical disciplines
	3. Virtual clinical encounters	Participants met and conversed with a physician and one of their patients
	4. Book club discussions	Discussed ‘No Apparent Distress’ by Rachel Pearson, MD; discussions were led by assigned PASS participants
	5. Didactics	Topics presented included: child abuse and advocacy, cultural competency, diversity in biomedical science, imposter syndrome, and trauma chaplaincy
	6. Virtual ‘field trips’	These included a virtual visit to the Philadelphia Museum of Art for art observation training and a virtual culinary medicine experience
Near-peer Mentoring/ Facilitation	1. Facilitation of weekly team-building activities	Served to intentionally foster the cohesion of the cohort of participants
	2. Facilitation of weekly group reflections on the PASS experiences	Enabled an opportunity to revisit, process, and contextualize the various program experiences
	3. Facilitation of Podcast discussions	The medical students provided framing and context to understand the broader significances of the podcasts
	4. 1-on-1 meetings with PASS participants	A critical element of the advising/coaching provided by the program
	5. Hosting of ‘Game Nights’	The Game Nights were opportunities for the PASS participants to come together for fun activities outside of the required programing
Advising/ Coaching	Each participant had a 1-on-1 meeting with one of the medical student coordinators, the program staff coordinator and program faculty director	The goal was to enable supportive relationships that facilitate participants’ transition to medical school and which can be leveraged in the future

In addition, the medical student coordinators hosted ‘Game Nights’ in which the participants could come together outside of the required scheduled programing on evenings and weekends for fun activities. The schedule of programing for the first two weeks is presented in the S1 Supplemental Information. Video conferencing was provided using the Zoom platform.

### Program assessments

#### Data acquisition

Participants completed a pre-program survey that included 5-point Likert-style and open-ended questions to determine baseline expectations as well as self-assessments of their research skills, familiarity with the physician identity, and preparedness to be a PSOM student. Students subsequently completed a post-program survey using 5-point Likert-style and open-ended questions to evaluate self-reported changes in research competence, sense of physician identity and preparedness for PSOM as well as the effectiveness of the program in achieving its goals. Finally, each event or activity was evaluated for its educational value, participant engagement, and overall quality by the participants using 5-point Likert-style questions and free text comments (see S2 Supplemental Information for the surveys). All surveys were anonymous. At the start of the program, participants were informed that the data would be collected anonymously and used for program evaluation and improvement. Further, throughout the program, participants were encouraged to complete the surveys to provide their honest feedback to the program team.

#### Data analysis

Mixed method approaches were used to analyze all survey data collected from the participants. Likert-style question results and other quantitative question results were analyzed using calculations of means and standard deviations (SD), using Microsoft Excel and R (Version 3.5.1). Percentages of ‘yes/no’ questions and gender differences were calculated. Free response question entries were qualitatively analyzed independently by three authors (CZ, CO, HD), searching for common themes across the participants’ responses and using a qualitative content analysis with inductive reasoning. This study was reviewed and approved by the Institutional Review Board of the University of Pennsylvania, and was deemed to be exempt given minimal risk to participants.

## Results

### Participant demographics

The 15 participants of PASS 2020 were undergraduate students from 9 institutions (5 institutions are historically black colleges and universities or HBCUs) across 5 different states. Participants represented 10 different majors ranging from Mathematics to Sports Medicine, with a majority of students studying Biology or Biology-related majors. Participants are all URM, with 11 students self-identifying as Black/African American, 3 students self-identifying as Hispanic/Latinx, and 9 students self-identifying as FGLI. Six participants were returning students who had participated in the in-person PASS program the previous summer of 2019, while 9 students were first-time participants of the program. A summary of program participant characteristics is presented in Table 3.

**Table 3.** Participant characteristics

Characteristics	Participants Number (%) (N = 15)
Gender (Self-identified)	
Female	9 (60.0%)
Male	6 (40.0%)
Ethnicity (Self-identified)	
Asian	1 (6.7%)
Black/African American	11 (73.3%)
Hispanic/Latinx	3 (20.0%)
FGLI Identity (Self-identified)	
First-generation	2 (13.3%)
First-generation and low-income	3 (20.0%)
Low-income	4 (26.7%)
Neither/Prefer not to answer	6 (40.0%)
Returning vs. First-Year Participant	
Returning	6 (40.0%)
First-Year	9 (60.0%)
Participants’ College/University	
Bryn Mawr College	1 (6.7%)
Haverford College	1 (6.7%)
Howard University	1 (6.7%)
Morehouse College	1 (6.7%)
Oakwood University	3 (20.0%)
Princeton University	2 (13.3%)
Spelman College	1 (6.7%)
University of Pennsylvania	3 (20.0%)
Xavier University of Louisiana	2 (13.3%)
Participants’ Undergraduate Major	
Biology/Biology-Related	10 (66.7%)
Computer Science	1 (6.7%)
Mathematics	1 (6.7%)
Engineering	1 (6.7%)
Psychology	1 (6.7%)
Sports Medicine	1 (6.7%)

### Assessments of program content and activities

Program activities/events were individually rated on a 5-point Likert scale (1 = poor, 5 = outstanding) by participants for their educational value, level of participant engagement during the activity and overall quality. For the entire group of 40 program activities/events, their mean educational value was rated at 4.33 (SD = 0.78); mean level of participant engagement during activities was rated at 4.38 (SD = 0.70); and mean overall quality of activities was rated 4.41 (SD = 0.68). The mean response rate for each activity or event was 71.5%. Participants’ qualitative comments on the various categories of activities were consistent with these high ratings and are summarized in the S3 Supplemental Information.

### Achieving the goals of the program

Participants were variously queried using a 5-point Likert scale (1 = strongly disagree/poor, 5 = strongly agree/outstanding) about the success of the program in achieving its explicit goals (Table 4, Sections A). Respondents scored the statements, ‘The PASS program accomplished all five of its stated goals’ at 4.73 (SD = 0.46), and ‘I would recommend the PASS program to my peers’ at 4.87 (SD = 0.35) indicating that the virtual formatting enabled PASS to achieve its program goals. Quantitative and qualitative data for each of the 5 goals are further discussed.

**Table 4.** Summary of participant responses to Likert-style questions on their PASS experience*

Item	Pre-Program (N = 15)	Post-Program (N = 15)	P value (for weighted means)
	Weighted Mean (SD)	Weighted Mean (SD)	
Section A			
The PASS program accomplished all five of its stated goals for the summer		4.73 (0.46)	
I would recommend the PASS program to my peers		4.87 (0.35)	
Section B			
I would rate my knowledge and understanding of what it means to be a physician as	3.13 (0.52)	4.00 (0.38)	< 0.001
Compared to the start of the program, my knowledge and understanding of what it means to be a physician increased significantly by the end of the program		4.60 (0.51)	
Section C			
I would rate my competence in doing research as	3.07 (0.70)	3.53 (0.52)	0.007
Compared to the start of the program, my competence and confidence in doing research increased significantly by the end of the program		4.33 (0.62)	
Section D			
I would rate the quality of the relationship between my research PI and as		4.40 (0.51)	
I would rate my interest in my research project as		4.07 (0.70)	
Section E			
There was a sense of community and cohesion among the PASS students at the end of the program		4.67 (0.49)	
I would rate the quality of the relationship between myself and the medical student coordinators as		4.33 (0.82)	
I would rate the quality of the relationship between myself and the program coordinators as		4.27 (0.70)	
I would rate the quality of the relationship between myself and the program director as		4.27 (0.70)	
Section F			
I would rate my preparedness to be a PSOM student as	3.27 (0.88)	3.93 (0.59)	0.003
Compared to the start of the program, I feel better prepared to be a Perelman student		4.13 (0.52)	

*1 = strongly disagree/poor, 2 = disagree/fair, 3 = neutral/good, 4 = agree/excellent, 5 = strongly agree/outstanding.

#### Development of physician identity and professionalism

Respondents’ self-reported knowledge/understanding of what it means to be a physician increased significantly at the end compared to the start of the program (Table 4, Section B, p < 0.001). Further, mean respondents at the end of the program ‘strongly agreed’ (4.6, SD = 0.51) with the statement, ‘Compared to the start of the program, my knowledge and understanding of what it means to be a physician increased significantly by the end of the program’ (Table 4, Section B).

Participants, before and after the program, provided qualitative comments about their understanding of what it means to be a physician. Three themes were noted in the pre-program responses of the participants. First, respondents described physicians in terms their actions and work, with most participants referencing physicians as providers of medical care. Participants also identified physicians as socially engaged (‘[helping] change my community for the better’); advocates for patients (‘advocating for those whose voice is silenced’), and translators of biomedical science research into care (‘[bringing] science and research to a much more personal and human level’). Second, participants spoke of physicians as having a package of humanistic qualities including, possessing a willingness to ‘sacrifice [one’s] needs for someone else’s’ as well as being ‘humble,’ ‘compassionate and supportive,’ and ‘always willing to do what they can to help.’ Finally, respondents referenced the high level of competence required of physicians, emphasizing that the physician is someone who is ‘always learning and sharpening [their] clinical skills.’

While respondents continued to reference advocacy and clinical competence, their descriptions of physician identity following the program focused more explicitly on the relational qualities of being a physician. In this regard students commented:

“Being a physician is having an in-depth understanding of medicine but also an understanding of the audience you’re serving. After this program I’ve come to realize that having a connection with your patients [and] empathizing with them really makes the biggest difference not only in the patient’s health outcome but in their overall day to day life.”

“A physician should not only be technically competent and deeply committed to their craft, but they should realize that practicing excellent medicine includes taking into account the relational aspect. Overall, they should go above and beyond to provide excellent care, this includes what’s taught as medicine, but can also mean simply listening to what their patient is going through and caring about their whole being.”

#### Competence in doing research

Respondents’ self-reported competence in their ability to do research increased significantly after this summer of PASS (Table 3, Section C, p = 0.007), with the mean respondent ‘agreeing’ (4.33, SD = 0.62) with the statement, ‘Compared to the start of the program, my competence and confidence in doing research increased significantly by the end of the program’ (Table 4, Section C).

At the start of the program, participants desired to be more competent in their understanding of the processes of conducting research; in reading and understanding relevant scientific literature; and in their verbal and written scientific communication skills, which are highlighted in the following respondent comments:

“I wish to understand how to write a research paper, how to properly construct a successful experiment to test a hypothesis, and how to properly review and contribute to another scientist’s research.”

“I wish to gain more experience in writing scientific research papers, creating research procedures, and to be more familiar with technologies used in lab settings.”

“I want to improve my experimental design, my scientific writing, my presentation skills, and my grant applications.”

“I would like to be more competent in gathering and analyzing research data, critically thinking, and knowing how to approach a research problem.”

In terms of research skills, following the program, respondents indicated that the PASS experience increased their competence in ‘acquiring data, effectively analyzing data, and accurately presenting data;’ ‘improved research paper comprehension, teamwork skills, and presentation skills;’ made them ‘better at reading papers, managing data and asking questions about things I don’t know about;’ and enhanced their skill at ‘[synthesizing] large amounts of research information, understanding different research papers, and reaching out for assistance.’

#### Research mentor-mentee relationship

Respondents scored the statement, ‘I would rate the quality of the relationship between my research PI and I as’ at 4.40 (SD = 0.51) (Table 4, Section D), indicating that participants viewed their mentor-mentee relationship as excellent to outstanding. Consistent with this, mean respondents scored the statement, ‘I would rate my interest in my research project as’ at 4.07 (SD = 0.7), indicating excellent engagement with their project (Table 4, Section D). The strengths of these relationships were reflected in post-program comments:

“[My mentor] challenged me, but in an appropriate and respectful way. He asked me questions and set reasonable expectations that he wanted me to meet each week as I progressed. He helped me grow as a scientist and as a thinker.”

“My mentor has introduced me into clinical research, which I am really interested in now. She also encouraged me each time we met, which boosted my confidence when I was conducting my research.”

Post-program, respondents particularly noted that the required weekly one-on-one meetings with the mentors were especially helpful in fostering the mentor-mentee relationship:

“My research mentor did a great job of having weekly 1-on-1 meetings with me, where I was able to develop a relationship with him and he also helped me get to know the other members of the lab.”

#### Sense of community and cohesion

Respondents scored the statement, ‘There was a sense of community and cohesion among the PASS students at the end of the program’ at 4.67 (SD = 0.49) (Table 4, Section E). In post-program comments, respondents cited the team-building exercises, the podcast and book club discussions and the Game Nights as experiences that contributed to the development of this strong sense of community and group cohesion. Participants also indicated that the sense of community and group cohesion were fostered by the presence of the medical student mentors and the program coordinator at program sessions as well as by the one-on-one meetings with program staff. As one student noted, ‘The individual meetings were really helpful. Also, having the [medical student coordinators] with us every day really allowed us to get to know each other well.’ These comments are consistent with the mean respondents scoring their relationships with the medical student mentors (4.42, SD = 0.49), program coordinator (4.31, SD = 0.70), and program director (4.31, SD = 0.70) as excellent to outstanding (Table 4, Section E).

#### Transition into medical school

Compared to the start of the program, respondents’ sense of preparedness to be a PSOM student increased significantly after participating in the program (Table 3, Section F, p = 0.003). Further, the mean respondent ‘agreed’ (4.13, SD = 0.52) that ‘compared to the start of the program, I feel better prepared to be a Perelman student’ (Table 3, Section F).

Students were queried at the beginning of the program about their greatest concerns regarding their preparedness to be a medical student at PSOM. The pace and workload of medical school were the concerns most often referenced by the participants. Representative comments included:

“I don’t think someone is ever completely prepared for medical school but I do want to be ready for the water coming out of the fire hydrant as much as possible.”

“I heard from several doctors and medical students how much material is covered in medical school and how much time they have to spend studying. I feel a little intimidated by the difficulty of medical school.”

Other concerns that were expressed included the cost of medical education (‘How I am going to pay for school and life expenses’); status as a racial minority (‘[adapting to] being a double minority in a predominantly white institution and profession’); and feelings of intimidation in attending a prestigious institution such as PSOM (‘Feeling empowered enough to see myself as a Perelman student’).

Participant comments after the program indicated that the PASS experience helped, at least in part, to alleviate these concerns as reflected in the following comments:

“I was concerned about paying for medical school and being able to adapt to the fast-paced environment, but I feel a little more comfortable with both.”

“I feel more confident in that when I encounter any problems during my time at Perelman, I have resources and people to connect with that will support me.”

“The discussion about imposter syndrome really helped to reassure me about this natural occurring feeling and that I am fit and worthy of being a medical student at Perelman.”

Despite fears and concerns, participants following the program were excited for the opportunities in medical school to connect with their peers and with patients, and to further develop their identities and skills as future physicians. As one student commented, ‘I am really looking forward to honing into how I want to be a physician … I am also looking forward to continuing relationships with PASS students and creating new relationships with other students.’

### Limitations of the virtual programing

Despite being well-received, there were challenges and limitations to the delivery of PASS using a virtual format. The first were technical. Because of a lack of a consistently stable internet connection, or weather-related disruptions in electrical power, some students missed parts of a session, were unable at times to contribute fully to a session and/or were disrupted while making a presentation.

Second, participants in their qualitative comments indicated a continued desire for on-campus experiences such as visiting the student-led community clinics, shadowing physicians, hands-on research in a bench lab, and physically engaging the other PASS students, program staff and research mentors. As one student who commented on a virtual versus an in-person experience said, ‘[I missed] shadowing [opportunities], physically having access to the campus and the surrounding community, being able to live with my peers and getting to know them in person.’ Even as respondents indicated that this cohort of PASS students achieved a high level of cohesion and community, they also acknowledged that the ‘in-person experience is irreplaceable to a degree’ and that ‘it would have been easier to get to know my fellow PASS scholars if we lived together.’

During the program, feedback was solicited from the participants to make real-time adjustments. We were particularly attentive to ‘Zoom fatigue’ [17–19]), which began to set in during the later weeks of the program, and so real-time adjustments were made in the timing, formatting, nature and amount of some of the program content to address this issue.

In addition to the real-time feedback, respondents also provided feedback in the post-program survey on the various program activities and events to enhance future iterations of the program. This included specific suggestions regarding the facilitation, duration and/or content of the programing, which are further highlighted in S3 Supplemental Information.

## Discussion

Racial and ethnic minorities continue to be underrepresented in medical school matriculants and in the physician-workforce, with this underrepresentation being particularly dramatic for Black males [1–5]. Efforts to address these disparities have led to the establishment of summer pre-medical enrichment programs targeting URM undergraduate students [6–16]. These programs, typically conducted on-site, at large research-focused medical schools and centers, have sought to enhance the competitiveness of these disadvantaged students for medical school admission and/or their preparedness for success once they have matriculated. This study contributes to the published literature on these programs by describing the initial outcomes of the virtual offering of a summer enrichment program for pre-medical URM students.

There are a number of processes that may disproportionately disadvantage URM students in either gaining admission to medical school or, once matriculated, getting off to a strong start [2,3]. First, these URM students are more likely to come from families that fall into a low socioeconomic status, or from which they are the first to go to college and/or medical school [20, 21]. This may, in turn, mean a lack of knowledge and experiences that facilitate admission and then transition to medical school [20–23]. Second, URM students aspiring for a career in medicine often lack meaningful exposure to physician role models with whom they identify with racially, ethnically or experientially and/or from whom they can draw inspiration and mentoring [2,24]. Third, given their minority status, it is not uncommon for URM students to experience loneliness and a sense of isolation, particularly early on in their medical school careers [25,26]. Finally, URM individuals may experience destabilizing psychological processes, such as performing to dispel racial stereotypes [27], stereotype threat [28,29], imposter syndrome [30,31] and other forms of minority stress [32,33].

PASS was developed to address these issues, which are reflected in its five interconnected program goals. The program seeks to facilitate participants’ successful transition to medical school by fostering an understanding of physician identity, competence in doing research, a strong research mentor-mentee relationship and a sense of community, that together enable student confidence and self-efficacy as well as establish affirming relationships that can be drawn on for future support. Among respondents, this virtual offering of PASS was seen as very successful in achieving the goals of the program. Specifically, respondents rated the educational value, participant engagement and quality of programing as excellent to outstanding and strongly agreed that PASS had achieved its program goals and thus would recommend it to their peers (Table 3, Section A).

As a result of this virtual offering of PASS, participants acquired knowledge in two domains that is likely to be helpful to them as they apply to and then subsequently progress in medical school. First, they matured in their understanding of what it means to be a physician (Table 3, Section B) [34,35]. Participants entered this offering of the program with a core understanding of physicians that centered on the provision of care, clinical competence and the possession of humanistic qualities, which grew to more explicitly emphasize the critical interpersonal and relational skills of being a physician and their importance to the doctor-patient relationship. Second, participants reported increased competence in doing research with respect to their design, analytical and presentation skills (Table 3, Section C) [36]. We believe these learning experiences help to address some of the knowledge deficits that might be associated with a URM student’s FGLI status.

Another important outcome were the relationships that students were able to develop during this virtual programing of PASS. Participants’ relationships with their research mentors and program staff (medical student coordinators, program coordinator and program director) were all rated as excellent to outstanding at the end of the program (Table 3, Sections D and E). Further, this cohort of PASS participants attained a deep sense of community and cohesion (Table 3, Section E). Both participant comments and the anecdotal experiences of the program team, suggest that the success in making this offering of PASS a relationship building experience for the participants was in part the result of the daily, yet varied interactive events and activities, active participant engagement in each session, and the frequent contact with program coordinators, staff and director. The program’s ability to foster these relationships enables the PASS participants to start medical school with a network of peer, near-peer and staff/faculty relationships already in place that they can turn to for community, support and advice to mitigate the isolation and loneliness that UIM medical students often experience [25,26].

Finally, the with respect to the transition to medical school, participants felt significantly more prepared to be a PSOM student after this virtual offering of PASS (Table 3, Section F). While not all concerns/fears were completely alleviated, following this summer of PASS, participants looked forward to medical school with excited anticipation. This outcome is likely the result of the combined effects of the didactics targeting specific concerns and fears (e.g., the imposter syndrome and diversity in health care) as well as the learning experiences and relationships they developed.

There is a growing body of literature that speaks to both the power as well as pitfalls of virtual learning, which has become essential during the COVID-19 pandemic [37,38]. Thus, while this virtual iteration of PASS was successful in achieving its goals, and was well-received by the participants, there were on-campus experiences participants yearned for, including direct clinical engagement with patients and clinicians, getting to know Philadelphia first-hand, and hands-on bench laboratory experiences. Further, although this virtual cohort achieved a high degree of community and cohesion, there was still the sense that deeper levels of connection would have been reached if the students had been on campus living together. We were particularly attentive to ‘Zoom fatigue’ [17–19] and so adjustments were made in the latter half of the programing to address this issue. Thus, whether virtual programing is employed by design or by necessity for the delivery of a URM premedical summer enrichment program, it is essential to be flexible, open to real-time, participant feedback and to meticulously adhere to best practices that maximize the learning as well as the opportunities for interpersonal connection [17–19].

Our findings have implications, not only for the continuance of established programs such as PASS, but also for the wider availability of pre-medical summer enrichment programs that target disadvantaged students. First, in not having to pay for housing and facilities charges, along with potentially less administrative costs, virtual programing may make diversity efforts of this type more affordable to institutions who have been constrained in doing so because of the costs associated with on-campus programing. Second, the virtual programing allows for participation of a potentially wider and more regionally diverse pool of participants who would not be limited by issues of travel for on-campus programing. Finally, a virtual format allows for recruitment of research mentors beyond that of a single institution as well as cross-institutional partnerships that enable robust programing not possible if the institutions acted individually.

There are several limitations to this study that should be noted. First, this study involved a small number of participants at a single institution and thus may not be generalizable to other institutions with a different mix of students. Second, the assessments of the program were all based on self-assessments. Thus, going forward, it be will necessary to evaluate the impact of the PASS program with more objective measures. Third, the assessments were done immediately after the program. Future studies should therefore assess the durability of what was learned and how PASS impacted participants’ medical school experience. Finally, we cannot exclude a selection bias for participants who would be much more favorably disposed toward the program.

Despite these limitations, our data provide evidence that premedical summer enrichment programs targeting URM undergraduates, offered virtually, can continue to be effective during challenges such as the COVID-19 pandemic in pursuing their goal of increasing medical trainee and physician-workforce diversity.

## Supplementary Material

Supplemental Material
