# Supplementary material for: Penn Access Summer Scholars program: a mixed method analysis of a virtual offering of a premedical diversity summer enrichment program
Source: Med Educ Online. 2021 Mar 31;26(1):1905918. doi: 10.1080/10872981.2021.1905918 (PMC8018359; doi:10.1080/10872981.2021.1905918)
Supplement: Supplemental Material [file ZMEO_A_1905918_SM6294.zip › supplementary files/S1 Supplemental Information.docx]

**Penn Access Summer Scholars Program: A Mixed Method Analysis of a**

**Virtual Offering of a Premedical Diversity Summer Enrichment Program**

**S1 Supplemental Information**

**Penn Access Summer Scholars Program**

**Summer 2020**

**Schedule for Weeks 1 and 2**

**Week 1 (6/29-7/3)**

| **Component** | **Time** | **Monday (6/29)** | **Tuesday (6/30)** | **Wednesday (7/1)** | **Thursday (7/2)** | **Friday (7/3)** |
| --- | --- | --- | --- | --- | --- | --- |
| **Research** | 9:00-1:00 | - Online module for the week: Responsible Conduct of Research | Initial meeting with Mentor | Research-related activities | Research-related activities | **Off for July 4^th^ Holiday** |
| **Enrichment** | 1:00-2:00 | Orientation (Pt. 1) | Open | Didactic  (Child Abuse and Advocacy, Cindy Christian [Pediatrics]) | Discussion of Responsible Conduct of Research |  |
|  | 2:00-2:15 | Break | Break | Break | Break |  |
|  | 2:15-3:15 | Orientation (Pt. 2) | Career Narrative  (Cary Aarons, MD, [Surgery]) | Podcast Discussion | End-of-Week Reflection |  |
|  | 3:15-3:30 | Wrap-up | Wrap-up | Wrap-up | Wrap-up |  |
| **Preparatory/**  **Flexible/**  **1-on-1 Meeting**  **Time** | 3:30-5:00 | Podcast for the Week:  [Dispatch 2: Every Day is Ignaz Semmelweis Day](https://www.wnycstudios.org/podcasts/radiolab/articles/dispatch-2-every-day-ignaz-semmelweis-day)  [Dispatch 4: Six Feet](https://www.wnycstudios.org/podcasts/radiolab/articles/dispatch-4-six-feet) | Preparatory/Flexible Time | Preparatory/Flexible Time | Preparatory/Flexible Time |  |

**Week 2 (7/6-7/10)**

| **Component** | **Time** | **Monday (7/6)** | **Tuesday (7/7)** | **Wednesday (7/8)** | **Thursday (7/9)** | **Friday (7/10)** |
| --- | --- | --- | --- | --- | --- | --- |
| **Research** | 9:00-1:00 | - Online Module for the Week: (i) How to Review a Research Article; (ii) How to create journal club presentation - Preparation for presentation on “My Lab/Research Group | Research-related activities | Research-related activities | Research-related activities | Research-related activities |
| **Enrichment** | 1:00-2:00 | Team Building | Didactic  (Cultural Competency, Horace DeLisser, MD) | Discussion (i) How to Review a Research Article; (ii) How to create journal club presentation | My Lab/Research Group | Virtual Gallery Experience at the PMA |
|  | 2:00-2:15 | Break | Break | Break | Break | Break |
|  | 2:15-3:15 | Virtual Clinical Encounter  (Horace DeLisser, [Pulmonary Medicine]) | Career Narrative  (Nahla Khalek, MD [Obstetrics &Gynecology]) | Podcast Discussion | My Lab/Research Group | End-of-Week Reflection |
|  | 3:15-3:30 | Wrap-up | Wrap-up | Wrap-up | Wrap-up | Wrap-up |
| **Preparatory/**  **Flexible/**  **1-on-1 Meeting**  **Time** | 3:30-5:00 | Podcast for the Week:  [Dr. Gilmer and Mr. Hyde](https://www.thisamericanlife.org/492/dr-gilmer-and-mr-hyde) | Preparatory/Flexible Time | Preparatory/Flexible Time | Preparatory/Flexible Time | Preparatory/Flexible Time |
