# Supplementary material for: Penn Access Summer Scholars program: a mixed method analysis of a virtual offering of a premedical diversity summer enrichment program
Source: Med Educ Online. 2021 Mar 31;26(1):1905918. doi: 10.1080/10872981.2021.1905918 (PMC8018359; doi:10.1080/10872981.2021.1905918)
Supplement: Supplemental Material [file ZMEO_A_1905918_SM6294.zip › supplementary files/S2 Supplemental Information.docx]

**Penn Access Summer Scholars Program: A Mixed Method Analysis of a Virtual Offering of a Premedical Diversity Summer Enrichment Program**

**S2 Supplemental Information**

**Penn Access Summer Scholars Program**

**Summer 2020**

**Virtual Program Surveys**

**Virtual PASS Pre-Program Survey**

*Required

**I would rate my knowledge and understanding of what it means to be a physician as ***

Poor

Fair

Good

Excellent

Outstanding

Summarize your current understanding of what it means to be a physician in a few sentences.

Your answer

**I would rate my competence in doing research as ***

Poor

Fair

Good

Excellent

Outstanding

List three of the most important research skills you have.

What skills do you wish you to be more competent in?

**I feel prepared to be a Perelman medical student. ***

Strongly Disagree

Disagree

Neutral

Agree

Strongly Agree

What are your biggest concerns regarding your preparedness to be a medical student at Perelman?

**I understand the five main goals of the PASS program. ***

Strongly Disagree

Disagree

Neutral

Agree

Strongly Agree

What aspects of the program are unclear or need more explanation?

**List three things you wish to learn or gain from your experience with the PASS program this summer. ***

**Virtual PASS Post-Program Survey**

*Required

**The PASS program accomplished all five of its stated goals for the summer.***

Strongly Disagree

Disagree

Neutral

Agree

Strongly Agree

Which objectives were best met?

Which objectives were poorly met?

**There was a sense of community and cohesion among the PASS students at the end of the program.***

Strongly Disagree

Disagree

Neutral

Agree

Strongly Agree

Which parts of the program contributed most to creating a sense of community and cohesion among the PASS students?

What would you add to increase the sense of community and cohesion among the PASS students?

**I would rate my knowledge and understanding of what it means to be a physician as ***

Poor

Fair

Good

Excellent

Outstanding

**Compared to the start of the program, my knowledge and understanding of what it means to be a physician has increased significantly by the end of the program.***

Strongly Disagree

Disagree

Neutral

Agree

Strongly Agree

Summarize your current understanding of what it means to be a physician in a few sentences.

**I would rate my competence in doing research as ***

Poor

Fair

Good

Excellent

Outstanding

**Compared to the start of the program, my competence and confidence in doing research have increased significantly***

Strongly Disagree

Disagree

Neutral

Agree

Strongly Agree

List three of the most important research skills that you now have.

What skills do you wish this program had taught you or you had more practice with?

**I would rate the quality of the relationship between my research mentor and I as***

Poor

Fair

Good

Excellent

Outstanding

What parts of the program allowed you to develop a quality relationship with your research mentor?

What parts of the program would you change to improve your research mentor-mentee relationships?

How has your research mentor helped you improve your research skills and how can they better help you in the future?

**I would rate my interest in my research project as ***

Poor

Fair

Good

Excellent

Outstanding

How has your research project influenced your interest in medical research, research overall, and/or that field of medicine?

**I would rate the quality of the relationship between the medical student mentors and me as ***

Poor

Fair

Good

Excellent

Outstanding

**I would rate the quality of the relationship between the program coordinator and me as ***

Poor

Fair

Good

Excellent

Outstanding

**I would rate the quality of the relationship between the program director and me as ***

Poor

Fair

Good

Excellent

Outstanding

What parts of the program allowed you to develop a quality relationship with your mentors?

What parts would you change to improve your mentor-mentee relationships?

**Compared to the start of the program, I feel better prepared to be a Perelman student***

Poor

Fair

Good

Excellent

Outstanding

In what ways were your concerns regarding your preparedness to be a medical student at Perelman resolved or unresolved by the program?

What are your new concerns about medical school (if any)?

What are you looking forward to about medical school?

**I feel prepared to be a Perelman medical student. ***

Strongly Disagree

Disagree

Neutral

Agree

Strongly Agree

**I would recommend the PASS program to my peers ***

Strongly Disagree

Disagree

Neutral

Agree

Strongly Agree

**(For returning students) I think the overall educational value and quality of the virtual PASS program is similar to the in-person PASS experience ***

Strongly Disagree

Disagree

Neutral

Agree

Strongly Agree

What parts of the in-person experience were missing and were essential to your experience and your learning in the program?

What parts of the virtual experience added to the program?

**List the three most important things you learned or gained from doing the PASS program this summer***

.

**Virtual PASS Post-Event/Activity Feedback Survey**

*Required

**I would rate the educational value of the event/activity as***

Poor

Fair

Good

Excellent

Outstanding

**I would rate the level of engagement with student preceptors and classmates during the event/activity as***

Poor

Fair

Good

Excellent

Outstanding

**I would rate the overall quality of the event/activity as***

Poor

Fair

Good

Excellent

Outstanding

**Comments/suggestions/questions:**
