# Supplementary material for: Penn Access Summer Scholars program: a mixed method analysis of a virtual offering of a premedical diversity summer enrichment program
Source: Med Educ Online. 2021 Mar 31;26(1):1905918. doi: 10.1080/10872981.2021.1905918 (PMC8018359; doi:10.1080/10872981.2021.1905918)
Supplement: Supplemental Material [file ZMEO_A_1905918_SM6294.zip › supplementary files/S3 Supplemental Information.docx]

**Penn Access Summer Scholars Program: A Mixed Method Analysis of a Virtual Offering of a Premedical Diversity Summer Enrichment Program**

**S3 Supplemental Information**

**Summary of participants qualitative comments on the various activities of the PASS program**

|  | **Activity** | **Summary of Student Comments** |
| --- | --- | --- |
| **1** | Book Club Discussions | Participants valued the book club discussions, not only for the insightful conversations led by their peers, but also for the smaller groups of 7-8 students to 1 student mentor ratio for each discussion. Students reported feeling more comfortable and being able to speak up more in the smaller group. |
| **2** | Career Narratives | Participants enjoyed that while the physician speakers were prepared for their sessions, they also spoke freely in casual conversation style about their journeys to medicine. Students commented that these speakers were inspirational and motivational. |
| **3** | Clinical Encounters | Participants emphasized the importance of witnessing doctor-patient relationships and described the experiences as heart-warming. These sessions also had the most reported technical difficulties. |
| **4** | Didactics | Participants found the didactics to be informative. There was also a suggestion for more time be allotted for the discussion of diversity issues. |
| **5** | Field Trips | The two field trip sessions were well-loved by students, but students expressed that more time should be allotted to these sessions. |
| **6** | Game Nights | These were well-received, with respondents expressing a desire for more time for participants to come together outside the scheduled events |
| **7** | Podcast Discussions | Participants appreciated the mixture of podcasts on heavy ethical problems to more lighthearted questions. They also found that a mixture of guided-questions and comments by the students-mentors with open-ended conversation were helpful to promote thought-provoking discussion. |
| **8** | Research and journal club presentations | Students thought the discussion aspects of these research lectures and journal clubs were engaging, although the presentations by some of the students were repetitive of their previous presentations. Some content was not helpful or informative and could have been taught in a shorter time-span. |
| **9** | Reflection sessions | Students enjoyed these sessions as a time to hear other students’ thoughts, however, the reflection questions as the program progressed were felt to be repetitive. |
| **10** | Team-building sessions | Participants were receptive to both online games as well as creative team activities |
